# First Molecular Identification of *Rickettsia aeschlimannii* and *Rickettsia africae* in Ticks from Ghana

**DOI:** 10.4269/ajtmh.22-0753

**Published:** 2024-01-30

**Authors:** Janice A. Tagoe, Seth O. Addo, Mba-tihssommah Mosore, Ronald E. Bentil, Bright Agbodzi, Eric Behene, Danielle Ladzekpo, Charlotte A. Addae, Shirley Nimo-Painstil, Anne T. Fox, Langbong Bimi, Courage Dafeamekpor, Allen L. Richards, Andrew G. Letizia, Joseph W. Diclaro, Samuel K. Dadzie

**Affiliations:** ^1^ Department of Parasitology, Noguchi Memorial Institute for Medical Research, College of Health Sciences, University of Ghana, Accra, Ghana;; ^2^ U.S Naval Medical Research Unit No. 3, Ghana Detachment, Accra, Ghana;; ^3^ Department of Animal Biology and Conservation Science, University of Ghana, Accra, Ghana;; ^4^ Ghana Armed Forces, Greater Accra, Ghana;; ^5^ Department of Preventive Medicine and Biostatistics, Uniformed Services University of the Health Sciences, Bethesda, Maryland;; ^6^ Infectious Diseases Directorate, Naval Medical Research Center, Silver Spring, Maryland;; ^7^ Navy Entomology Center for Excellence, Jacksonville, Florida

## Abstract

The threats from vector-borne pathogens transmitted by ticks place people (including deployed troops) at increased risk for infection, frequently contributing to undifferentiated febrile illness syndromes. Wild and domesticated animals are critical to the transmission cycle of many tick-borne diseases. Livestock can be infected by ticks, and serve as hosts to tick-borne diseases such as rickettsiosis. Thus, it is necessary to identify the tick species and determine their potential to transmit pathogens. A total of 1,493 adult ticks from three genera—*Amblyomma*, *Hyalomma*, and *Rhipicephalus*—were identified using available morphological keys and were pooled (*n* = 541) by sex and species. *Rickettsia* species were detected in 308 of 541 (56.9%) pools by genus-specific quantitative polymerase chain reaction assay (Rick17b). Furthermore, sequencing of the outer membrane protein A and B genes (*ompA and ompB*) of random samples of *Rickettsia*-positive samples led to the identification of *Rickettsia aeschlimannii* and *Rickettsia africae* with most *R. africae* DNA (80.2%) detected in pools of *Amblyomma variegatum*. We report the first molecular detection and identification of the rickettsial pathogens *R. africae* and *R. aeschlimannii* in ticks from Ghana. Our findings suggest there is a need to use control measures to prevent infections from occurring among human populations in endemic areas in Ghana. This study underscores the importance of determining which vector-borne pathogens are in circulation in Ghana. Further clinical and prevalence studies are needed to understand more comprehensively the clinical impact of these rickettsial pathogens contributing to human disease and morbidity in Ghana.

## INTRODUCTION

Ticks are currently considered second in importance to mosquitoes as human infectious disease vectors in the world.^1^ They are the vectors of the spotted fever group rickettsiae,^2^^–^^4^ which have been identified as significant agents in human tick-borne infections worldwide. In particular, African tick-bite fever (ATBF) has raised concerns beyond the continent because it is considered one of the leading causes of fever among travelers returning from sub-Saharan Africa.^5^^,^^6^

African tick-bite fever is caused by the pathogen *Rickettsia africae* and is transmitted by *Amblyomma variegatum* and *Amblyomma hebraeum*, the predominant and aggressive tick species in Africa.^7^ The majority of ATBF cases are reported in South Africa (> 80%), with *R. africae* infection transmitted by *A. variegatum* approximately 70% of the time and *A. hebraeum* approximately 30%.^8^ However, *R. africae* is reported to be widely distributed across the continent, as it has either been isolated or detected by polymerase chain reaction (PCR) in Kenya, Chad, Burundi, Mali, Senegal, Niger, Sudan, and in most South African countries.^8^^,^^9^ Despite the increasing information available on ticks in other parts of the world, and extensive work in some parts of West Africa such as Senegal^9^ and Nigeria,^10^^,^^11^ there are limited published data on tick species, their presence, prevalence, distribution, and the tick-borne pathogens they transmit in Ghana.

In Ghana, many household pets and livestock are commonly infested with ticks, making it a possible risk zone for ATBF.^12^ However, to date, *R. africae* has not been identified in ticks from Ghana; *Rickettsia felis* has been the only *Rickettsia* species found in Ghana and was obtained from blood samples of febrile children in the Ashanti region of Ghana.^13^ Recent studies of ticks collected in different areas of Ghana have shown that *A. variegatum* ticks are predominant,^14^^,^^15^ suggesting that *R. africae* may be present but has yet to be detected. Data from this study will be beneficial in guiding force health protection for both the U.S. and Ghanaian Armed Forces, as well as in enhancing global health security countermeasures. This study sought to characterize *A. variegatum* and other tick species, and to determine their potential role in the transmission of pathogenic *Rickettsia* species in Ghana.

## MATERIALS AND METHODS

### Tick sampling, identification, and processing.

**Figure 1.**
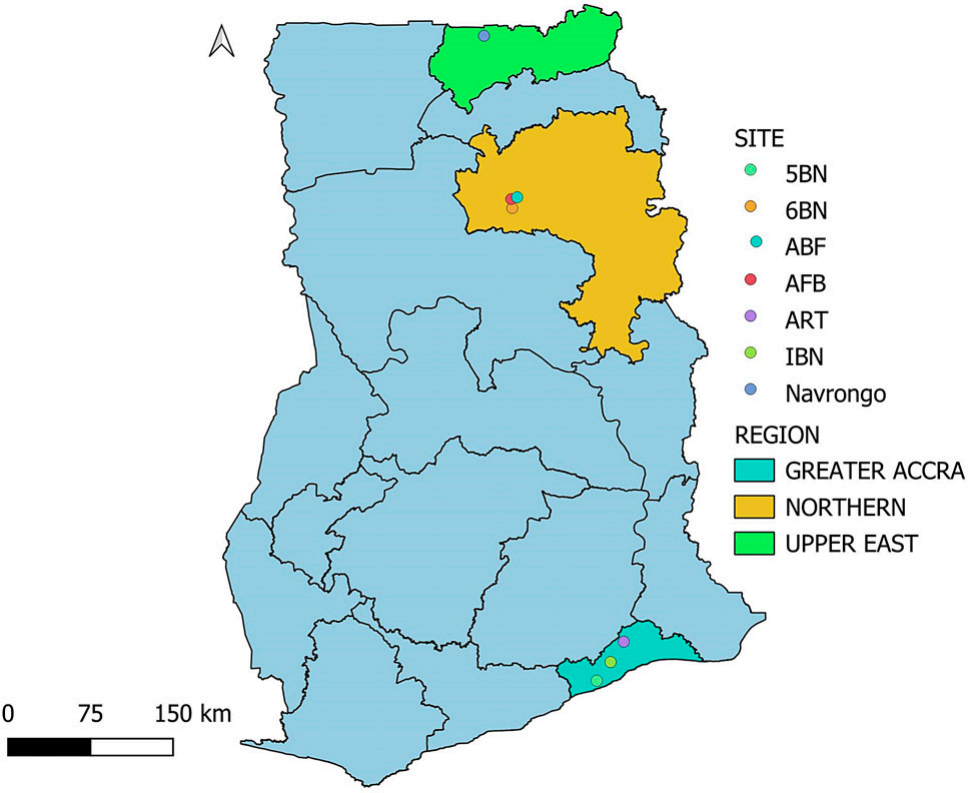
A map of Ghana indicating tick sampling sites. The sectors comprise Navrongo, the Sixth Battalion Infantry (6BN), Air Force Base (AFB), Air-Borne Force (ABF), Fifth Battalion Infantry (5BN), First Battalion Infantry (1BN), and Army Recruit Training School (ARTS). The map was developed using QGIS (version 3.30.3), https://www.qgis.org/en/site/forusers/download.html.

Adult ticks were collected from cattle during a tick survey carried out between August 2017 and March 2018 from seven sites in the southern and northern sectors of Ghana (Figure 1). Ticks were identified using morphological keys^16^ and pooled (five individuals or less) according to species, sex, and collection site for bacterial nucleic acid detection.

Nucleic acid was extracted using a QIAamp Viral RNA Mini Kit^17^ (QIAGEN, Valencia, CA) following the manufacturer’s instruction without adding carrier RNA to maintain DNA content. The tick DNA preparations were screened by a genus-specific quantitative real-time polymerase chain reaction (qRT-PCR) assay (Rick17b), with primers targeting the gene encoding the 17-kDa antigen of *Rickettsia* DNA as described previously.^18^ *Rickettsia*-positive pools were tested by a species-specific qRT-PCR assay (RafriG) for *R. africae* as described previously.^19^ The negative control was nuclease-free water whereas the positive control was *Rickettsia* DNA from a field isolate. Random samples that were positive by genus-specific qRT-PCR were selected from different collection sites in the southern and northern sectors for further characterization using primers targeting the outer membrane protein A gene (*ompA*)^20^ and outer membrane protein B gene (*ompB*).^21^ The amplification products were purified using the QIAquick PCR Purification kit (QIAGEN) and were sequenced using the Applied Biosystem 3730XL (Applied Biosystems, Foster City, CA).

Sequences obtained from our study were used to query the National Center for Biotechnology Information (NCBI) database using the Basic Local Alignment Search Tool (BLAST) to retrieve reference sequences for comparison. Sequences were aligned using ClustalW implemented in MEGA X. Tree model inference and phylogeny were conducted simultaneously in IQ-TREE (version 1.6.1), executing 1,000 bootstrap replicates. Tree visualization was done in FigTree (version 1.4.4).

The infection rates in the tick pools were calculated using PoolScreen 2.0. (version 2.0.1).^22^

## RESULTS

A total of 1,493 adult ticks were collected, comprising 516 females (34.6%) and 977 males (65.4%). The engorged ticks could not be identified to the species level because some morphological features were not visible; these were *Rhipicephalus* spp. (*n* = 203, 13.6%) and *Rhipicephalus* (subgenus *Boophilus*) (*n* = 1, 0.1%). Five tick species were identified: *Amblyomma variegatum* (*n* = 1,092, 73.1%), *Rhipicephalus sanguineus* s.l. (*n* = 73, 4.9%), *Hyalomma truncatum* (*n* = 66, 4.4%), *Hyalomma rufipes* (*n* = 49, 3.3%) and *Rhipicephalus evertsi* (*n* = 9, 0.6%).

Of the 541 tick pools, *Rickettsia* spp. were detected in 308 (56.9%) (Table 1). Five pools (83.3%) of *H. truncatum* were positive for *Rickettsia* spp., with an infection rate of 76.0% (95% CI, 30.0–99.0). A significant difference (*P* > 0.001) was seen in *Rickettsia* infections across the sampling sites, tick sex, and tick species, with most infections in male *A. variegatum* collected from Air-Borne Force. Furthermore, *R. africae* was detected in 238 *Rickettsia*-positive pools (77.3%) by species-specific qRT-PCR. It was observed that 202 pools of *A. variegatum* (80.2%) were positive for *R. africae*, with an infection rate of 54.2% (95% CI, 47.8–60.7).

**Table 1:** Infection rates of *Rickettsia* spp. and *Rickettsia africae* among pooled tick samples

Tick species	No. of ticks	No. of tick pools tested	*Rickettsia* positive		*Rickettsia africae* positive	
			No. positive pools	Minimum infection rate (95% CI)	No. positive pools	Minimum infection rate (95% CI)
*Amblyomma variegatum*	1,092	362	252	43.2 (38.4–48.2)	202	54.2 (47.8–60.7)
*Rhipicephalus sanguineus* s. l	73	45	14	19.3 (10.4–31.1)	9	42.1 (20.8–68.2)
*Rhipicephalus* spp.	203	100	23	12.7 (7.9–19.0)	18	57.1 (36.5–77.1)
*Hyalomma truncatum*	66	6	5	76.0 (30.9–99.0)	2	29.3 (3.9–72.2)
*Hyalomma rufipes*	49	22	11	30.7 (15.6–49.6)	5	30.8 (10.4–58.9)
*Rhipicephalus evertsi*	9	5	2	22.5 (2.9–60.2)	1	29.3 (1.0–85.6)
*Rhipicephalus* (*Boophilus*) sp.	1	1	1	100	1	100
Total	1,493	541	308	–	238	–

**Figure 2.**
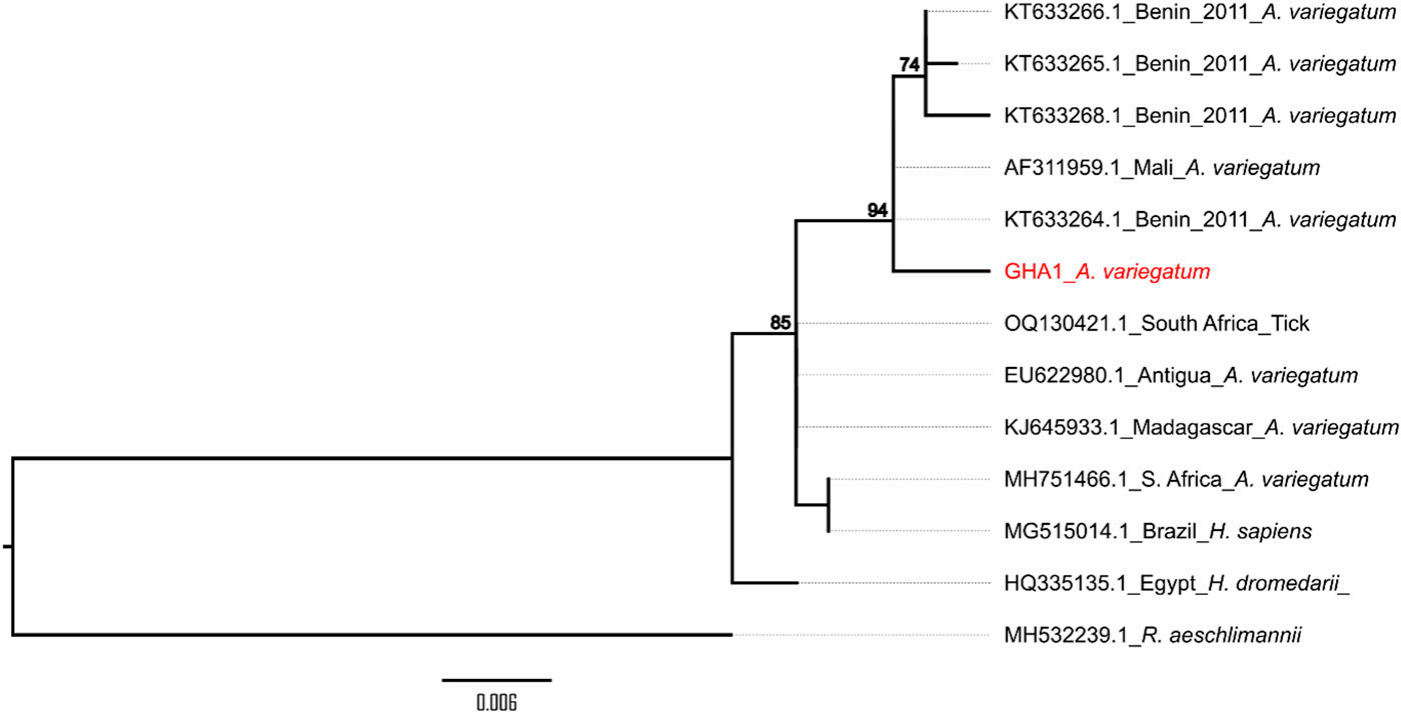
Phylogenetic analysis of the *Rickettsia africae* sequence from Ghana (red) and others from different geographic origins. The tree was constructed from a partial *ompA* gene segment (567 bp). Tree model inference and phylogeny were conducted simultaneously in IQ-TREE (version 1.6.1), executing 1,000 bootstrap replicates. The reference sequences included in the analyses are shown by their GenBank accession number, country of origin, and/or isolation date and host. Critical nodes are labeled with bootstrap values. The tree was visualized in FigTree (version 1.4.4), https://github.com/rambaut/figtree/releases.

**Figure 3.**
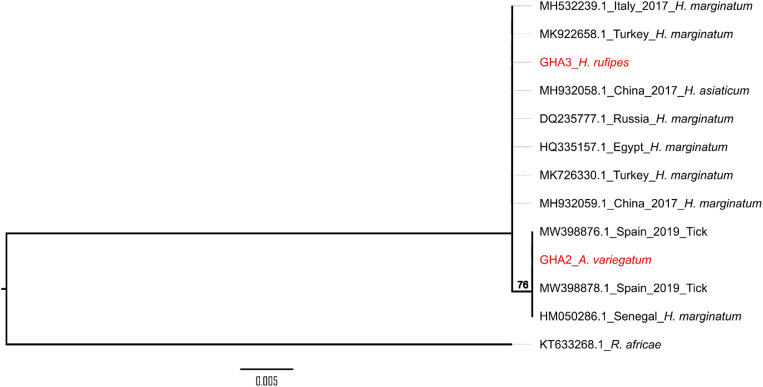
Phylogenetic analysis of two Ghana *Rickettsia aeschlimannii* sequences (red) and others from different geographic origins. The tree was constructed from a partial *ompA* gene segment (529 bp). Tree model inference and phylogeny were conducted simultaneously in IQ-TREE (version 1.6.1), executing 1,000 bootstrap replicates. The reference sequences included in the analyses are shown by their GenBank accession number, country of origin, and/or isolation date and host. Critical nodes are labeled with bootstrap values. The tree was visualized in FigTree (version 1.4.4).

In addition, 12 PCR products were sequenced successfully and compared with those available in the GenBank database using BLAST analyses. The sequences of *ompA*- and *ompB*-positive amplicons gave the same identification results. The BLAST search showed that one of the sequences was 99% identical to an *R. africae* isolate from Benin, and 11 sequences were 100% identical to *Rickettsia aeschlimannii* isolates from China and Spain (Figures 2 and 3).

The sequences from bacterial DNA preparations in our study were compared with other sequences in the NCBI database, and corresponding hit sequences were used to generate the phylogenetic trees shown in Figures 2 and 3 for *ompA*. The sequence from *R. africae* GHA1 clustered with isolates from Benin (KT633264.1, KT633266.1, KT633265.1, and KT633268.1) and Mali (AF311959.1). The *ompA* gene sequences from *R. aeschlimannii* GHA1 clustered with *R. aeschlimannii* from China (MH932058.1 and MH932059.1), Italy (MH532239.1), Turkey (MK922658.1 and MK726330.1), Russia (DQ235777.1), and Egypt (HQ335157.1). The other *R. aeschlimannii* (GHA2) *ompA* sequence clustered with isolates from Spain (MW398876.1 and MW398878.1) and Senegal (HM050286.1). The sequences selected were ≥ 98% similar to the *ompA* gene sequence of *R. aeschlimannii* Ghana 1 and 2 after the BLAST analysis. *Rickettsia aeschlimannii* (GHA1 and GHA2) detected in ticks in our study were not identical to each other. A single nucleotide change was observed in the *ompA* DNA sequence when sequence alignments were performed, and this could be responsible for the divergence observed between the two Ghana *ompA* sequences.

## DISCUSSION

Similar to previous studies in Ghana, *A. variegatum* was the predominant tick species.^23^^,^^24^ *Amblyomma variegatum*, an important vector in transmitting various rickettsial and viral pathogens, is infected with Crimean-Congo hemorrhagic fever^14^ and Dugbe viruses^15^ in Ghana. It was previously thought that *A. variegatum* was the only reservoir for *Rickettsia* spp. in sub-Saharan Africa.^3^ However, more recent studies have identified increased diversity in both *Rickettsia* spp. and the associated tick species harboring them.^25^^,^^26^ Our study demonstrates the same, with the identification of *R. aeschlimannii* and *R. africae* DNA in multiple tick species.

The prevalence of *Rickettsia* spp. in ticks collected from cattle in our study was greater than that reported from cattle in Zambia (18.6%)^27^ and Nigeria (12.5%),^11^ and from different animal species, including cattle from northern Senegal (5.8%).^28^ The high rate of infection observed in our study could be a result of the number of susceptible livestock present at the various sampling sites. The more livestock infected with *Rickettsia* spp., the greater the rate at which ticks will be infected during blood feeding and will potentially transmit to animal handlers. Further species identification of the *Rickettsia*-positive pools demonstrated a high prevalence of *R. africae* infection in the *A. variegatum* and *Rhipicephalus* species. Transovarial and trans-stadial transmission of *R. africae* has been demonstrated in *A. variegatum*.^29^ Thus, in the presence of cattle reservoirs, *A. variegatum* poses a significant risk to the human population. In Africa, although *R. africae* infection is common, it is rare to find reports of ATBF in indigenous people.^7^ This could be a result of chronic and recurrent exposure conferring some level of immunity. It could also be that cases are mild and unreported, or that effective diagnostic methods for *Rickettsia* are not available. However, ATFB is one of the most frequently reported causes of febrile illness among travelers returning from Africa.^9^ The most common clinical symptoms include fever, rash, headache, chills, lymphangitis, and fatigue.^30^ However, the nonspecific presentation can present as an undifferentiated febrile illness in travelers and individuals living in areas where the pathogen is endemic.

Sequencing analysis revealed that *R. aeschlimannii,* a pathogenic agent in the spotted fever group,^31^ was present in 11 of 12 samples. This pathogen was first identified and characterized after it was isolated from *Hyalomma marginatum* in Morocco.^32^ Since then, *R. aeschlimannii* has been identified frequently in other *Hyalomma* tick species in various West African countries, including Côte d’Ivoire, Nigeria, Senegal, Mali, and Niger,^25^^,^^33^^,^^34^ and is reported infrequently in *Amblyomma* and *Rhipicephalus* ticks.^3^ As in other areas of West Africa, our study identified *R. aeschlimannii* in *H. rufipes* ticks in Ghana. In addition, it provides evidence of *R. aeschlimannii* in *A. variegatum* and *Rhipicephalus* spp. Because the ticks assessed were collected from livestock, the presence of *R. aeschlimannii* may have been a result of the presence of the agent within the blood meal. This requires further blood meal analysis to determine hosts and/or reservoirs of the pathogen.

The pathogenicity of *R. aeschlimannii* in humans is not well understood, but based on limited reports of human infection, it appears to mimic Mediterranean spotted fever,^35^ with symptoms ranging from fever and sore throat to myalgias, maculopapular rashes, and acute hepatitis.^36^ Although no human infections with *R. aeschlimannii* have been reported in Ghana, this does not discount the circulation of this pathogen in the population, resulting from its documented presence in at least three tick species and the lack of routine testing in febrile patients.

This first reported molecular detection of *R. aeschlimannii* and *R. africae* in ticks collected in Ghana highlights the potential risk of infection and illness among animal handlers, within the community at large, and among travelers and deployed military personnel. Additional surveillance studies need to be performed to access the prevalence and distribution of ticks, the transmission of tick-borne pathogens, and their public health importance.

A limitation of the pooling method used in our study is that the identified *Rickettsia* species cannot be associated with individual tick species. Furthermore, pooling could have caused a reduction in the concentration of *Rickettsia* DNA, leading to false negatives. Engorged ticks that could not be identified to the species level but were subjected to pathogen screening could have introduced bias into the analysis.

## CONCLUSION

This study reports the dominance of *Amblyomma variegatum* ticks in sampled sites that harbored *Rickettsia* species of clinical significance to the U.S. and Ghanaian military, as well as to tropical medicine. Interestingly, for the first time in Ghana, our report identifies the presence of *R. aeschlimannii* and *R. africae*. However, confirming the vector and existence of these pathogens in Ghana is only the first step in determining the clinical impact of human disease in Ghana. Further studies assessing etiologies of undifferentiated fever are needed to determine the prevalence of the contribution of these rickettsial agents to human disease transmission in Ghana.
